# NADPH oxidase 2-derived reactive oxygen species signal contributes to bradykinin-induced matrix metalloproteinase-9 expression and cell migration in brain astrocytes

**DOI:** 10.1186/1478-811X-10-35

**Published:** 2012-11-23

**Authors:** Chih-Chung Lin, Hsi-Lung Hsieh, Ruey-Horng Shih, Pei-Ling Chi, Shin-Ei Cheng, Jin-Chung Chen, Chuen-Mao Yang

**Affiliations:** 1 Department of Anesthetics, College of Medicine, Chang Gung University and Chang Gung Memorial Hospital at Linkuo, Kwei-San, Tao-Yuan, Taiwan; 2 Department of Nursing, Division of Basic Medical Sciences, Chang Gung University of Science and Technology, Tao-Yuan, Taiwan; 3 Department of Physiology and Pharmacology and Health Aging Research Center, College of Medicine, Chang Gung University, 259 Wen-Hwa 1st Road, Kwei-San, Tao-Yuan, Taiwan

**Keywords:** Brain inflammation, Astrocytes, Bradykinin, Matrix metalloproteinase-9, NADPH oxidase, Reactive oxygen species

## Abstract

**Background:**

Matrix metalloproteinase-9 (MMP-9) plays a crucial role in pathological processes of brain inflammation, injury, and neurodegeneration. Moreover, bradykinin (BK) induces the expression of several inflammatory proteins in brain astrocytes. Recent studies have suggested that increased oxidative stress is implicated in the brain inflammation and injury. However, whether BK induced MMP-9 expression mediated through oxidative stress remains virtually unknown. Herein we investigated the role of redox signals in BK-induced MMP-9 expression in rat brain astrocytes (RBA-1 cells).

**Results:**

In the study, we first demonstrated that reactive oxygen species (ROS) plays a crucial role in BK-induced MMP-9 expression in cultured brain astrocytes (*in vitro*) and animal brain tissue (*in vivo*) models. Next, BK-induced MMP-9 expression is mediated through a Ca^2+^-mediated PKC-α linking to p47^phox^/NADPH oxidase 2 (Nox2)/ROS signaling pathway. Nox2-dependent ROS generation led to activation and up-regulation of the downstream transcriptional factor AP-1 (*i.e.* c-Fos and c-Jun), which bound to MMP-9 promoter region, and thereby turned on transcription of MMP-9 gene. Functionally, BK-induced MMP-9 expression enhanced astrocytic migration.

**Conclusions:**

These results demonstrated that in RBA-1 cells, activation of AP-1 (c-Fos/c-Jun) by the PKC-α-mediated Nox2/ROS signals is essential for up-regulation of MMP-9 and cell migration enhanced by BK.

## Background

Matrix metalloproteinases (MMPs) is a large family of zinc-dependent endopeptidases, which play an important role in the turnover of extracellular matrix (ECM) and pathophysiological processes [1]. In the central nervous system (CNS), MMPs, in particular MMP-9, have been shown to be involved in morphogenesis, wounding healing, and neurite outgrowth, [2]. Up-regulation of MMP-9 has been induced by various brain injuries, which may participate in the pathogenesis of brain diseases [3]. Moreover, cytokines and lipopolysaccharide (LPS) have been shown to induce MMP-9 expression and activity in culture rat brain astrocytes [4,5]. These studies demonstrated that MMP-9 may be involved in brain inflammation and injury.

Reactive oxygen species (ROS) are produced by various enzymatic and chemical processes or directly inhaled, including O_2_^-^, ·OH, and hydrogen peroxide (H_2_O_2_). The ROS at low level have physiological roles as signaling molecules in various cellular and developmental processes [6,7] and killing of invading microorganisms [8]. In contrast, recent report indicated that oxidative stress plays an important role in the progression of various diseases [8]. Moreover, ROS has been shown to interact with DNA, lipids, proteins, and carbohydrates that lead to cellular dysfunctions and inflammatory responses [7,9]. Under pathological conditions, many proinflammatory mediators like bradykinin (BK) induce expression of several inflammatory genes during brain injury by increasing ROS production [7,10]. Recently, increasing evidence attributes the neurodegenerative diseases such as Alzheimer’s disease (AD) to oxidative stress (generation of free radicals) that leads to brain inflammation during CNS pathogenesis [7,10,11]. Moreover, ROS also exert as a signaling factor mediated microglial activation induced by several proinflammatory mediators [12]. Although the effects of BK associated with ROS generation have been reported in several organ diseases [13], BK-induced ROS-mediated MMP-9 responses are not well characterized in rat brain astrocytes (RBA-1) cells.

BK and related peptides are elevated during brain trauma, stroke, and neuroinflammation [14]. Among brain cells, astrocytes possess a B2-type BK receptor which is a G protein-coupled receptor (GPCR) coupling to a G_q_ protein to activate PLCβ, phosphoinositide breakdown, Ca^2+^ mobilization, PKC, and ROS generation [15,16]. These signaling pathways regulate several cellular responses including proliferation, migration, and gene expression. Moreover, the 5’ region of MMP-9 promoters has been characterized to possess a series of functional enhancer element-binding sites such as NF-κB, Ets, and AP-1 [17]. BK has been shown to regulate expression of several genes through different transcription factors including AP-1 [18] and NF-κB [19]. In astrocytes, MMP-9 expression induced by various stimuli was mediated through activation of either NF-κB or AP-1 [20-22]. However, the molecular mechanisms of ROS associated with MMP-9 expression and cellular function (motility) induced by BK in RBA-1 cells are not completely defined.

Accordingly, BK may play an important role in regulation of ROS signal and specific gene expression, such as MMP-9 and enhancing inflammatory responses. The experiments were performed to investigate the molecular mechanisms of ROS signaling pathways involved in BK-induced MMP-9 expression in RBA-1 cells and an animal model. In the study, we found that BK induces MMP-9 expression via a ROS-dependent pathway. A Ca^2+^/PKC-α-dependent Nox2/ROS generation cascade contributes to AP-1 induction and activation, which is required for MMP-9 expression and cell motility in RBA-1 cells.

## Results

### BK induces MMP-9 expression via ROS-dependent manner

**Figure 1:**
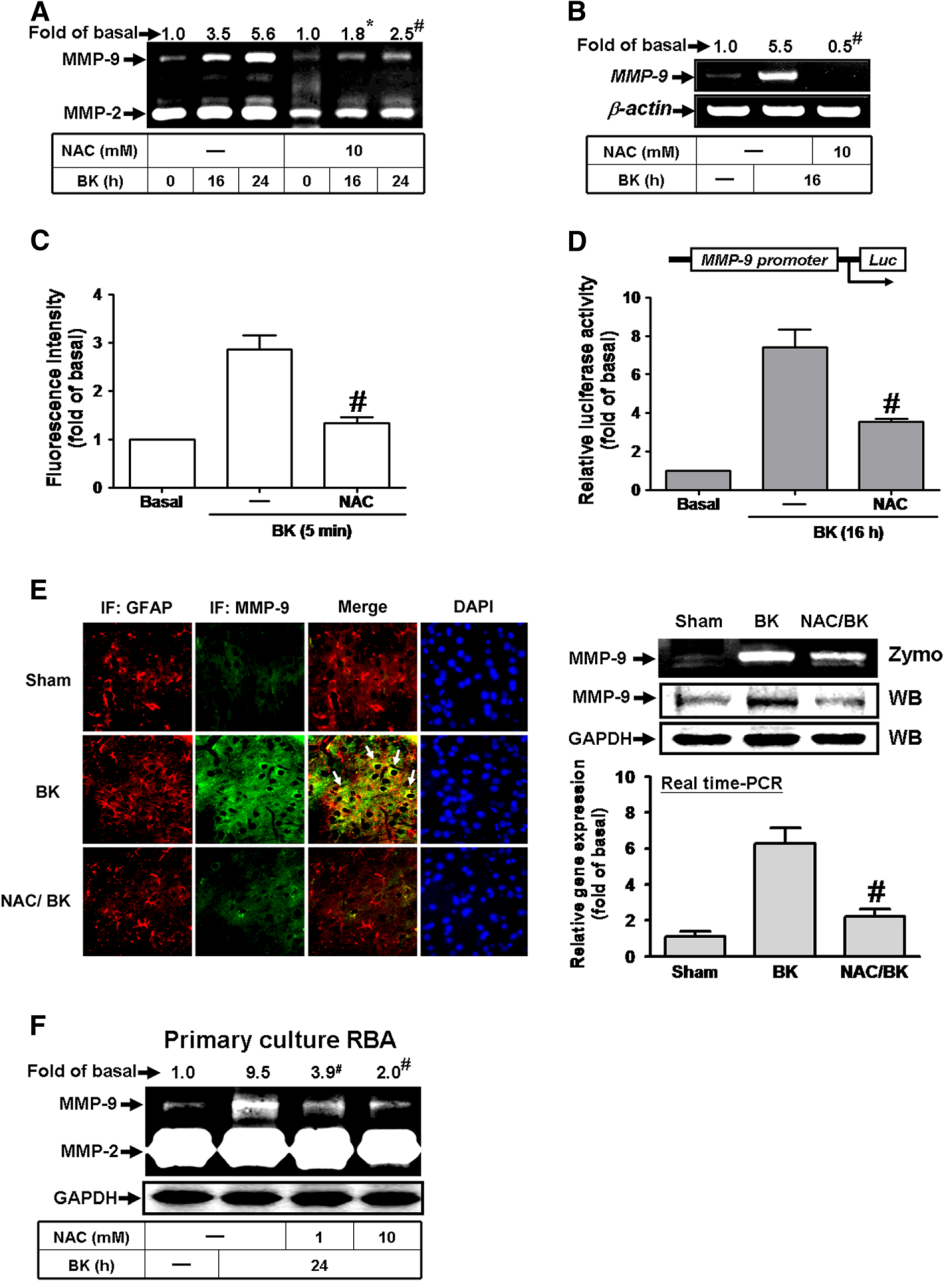
**ROS is a fundamental element during BK-induced MMP-9 expression in RBA-1 cells.** (**A**) Cells were pretreated without or with N-acetylcysteine (NAC, 10 mM) for 1 h before exposure to 10 nM BK for 24 h. The conditioned media were collected for zymographic analysis of MMP-9 expression and activity. (**B**) Cells were pretreated with or without NAC for 1 h before exposure to BK for 16 h. The total RNA was collected and analyzed by RT-PCR. (**C**) Cells were incubated with the DCF-DA (5 μM) for 45 min, followed by stimulation with 10 nM BK for 5 min in presence or absence of NAC. The fluorescence intensity of cells was determined. (**D**) Cells were transiently cotransfected with 0.9 μg of pGL-MMP9-Luc and 0.1 μg of pGal coding for β-galactosidase for overnight. Cells were treated with BK for 16 h in the absence or presence of NAC (10 mM) for 1 h before being harvested for measuring the luciferase and β-galactosidase activities*.* (**E**) Immunofluorescence staining for MMP-9 or GFAP in serial sections of the brain cortex tissue from vehicle-treated rat (Sham), BK-injected rat (BK), or NAC-pretreated rat (NAC/BK). The arrow indicates GFAP-positive cells overlapping with MMP-9 expression. DAPI was used to stain the nucleus. Image of fluorescence microscope, 400×. Similarly, the MMP-9 protein and mRNA of brain cortex tissues (Sham, BK, and NAC/BK) were analyzed by Western blot, zymography, and real-time PCR. (F) Rat primary astrocytes were isolated and cultured, and pretreated with or without NAC (1 or 10 mM) before exposure to BK for 24 h. The conditioned media and cell lysates were collected and analyzed by zymography and Western blotting. Data are expressed as the mean ± SEM (n = 3). ^*^P < 0.05; ^#^*P* < 0.01, as compared with the respective values of cells stimulated with BK only.

ROS have been shown to induce MMPs expression in various cell types [23]. To determine whether ROS participates in MMP-9 induction, a ROS scavenger (NAC) was used. Pretreatment with a NAC (10 mM) attenuated BK-induced MMP-9 protein and mRNA expression (Figure 1A, B). To explore BK-stimulated ROS generation and the efficacy of NAC, a ROS indicator (DCF-DA) was used. As shown in Figure 1C, BK-stimulated ROS generation was attenuated by pretreatment with NAC, demonstrating that NAC can efficiently scavenge ROS in these cells. To further investigate whether BK induces MMP-9 transcription activity via ROS-dependent manner, a rat MMP-9 promoter reporter-luciferase construct was used. The data showed that BK stimulated an increase of MMP-9 promoter activity which was attenuated by pretreatment with NAC (Figure 1D). These results suggested that ROS contribute to BK-induced MMP-9 expression via enhancing its gene transcriptional activity in RBA-1 cells.

We further confirmed whether BK-induced ROS-dependent MMP-9 expression also occurred in rat brain cortex tissue (*in vivo*). As shown in Figure 1E, the images of immunofluorescence staining showed that injection with BK induced MMP-9 expression as compared with that of control (sham), which was attenuated by pretreatment with NAC in rat brain cortex tissue. Consistently, pretreatment with NAC markedly attenuated BK-induced MMP-9 protein and mRNA expression (*in vivo*) determined by Western blot, gelatin zymography, and real-time PCR, demonstrating that BK induces MMP-9 gene expression via a ROS-dependent manner in *in vivo* studies. To further confirm BK-induced ROS-dependent MMP-9 expression in brain astrocytes, the rat brain primary astrocytes were isolated and cultured. As expected, pretreatment with NAC concentration-dependently inhibited BK-induced MMP-9 expression determined by gelatin zymography (Figure 1F). These results indicated that BK-induced ROS-dependent responses in RBA-1 cells, are similar to those of either animal model or rat primary cultured astrocytes. Thus, the following experiments were performed using RBA-1 cells throughout this study.

### Nox2-derived ROS generation contributes to BK-induced MMP-9 expression

**Figure 2:**
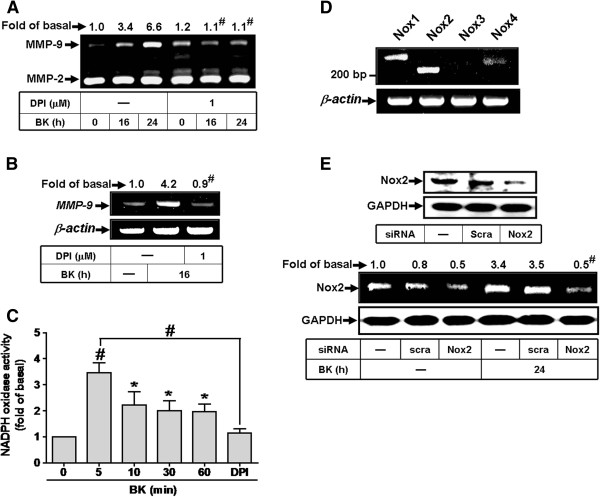
**NADPH oxidase-dependent ROS generation is involved in BK-induced MMP-9 expression in RBA-1 cells.** (**A**) Cells were pretreated without or with DPI (1 μM) for 1 h before exposure to 10 nM BK for the indicated time intervals. The conditioned media were collected for zymographic analysis of MMP-9 expression. (**B**) Cells were pretreated with or without DPI (1 μM) for 1 h before exposure to 10 nM BK for 16 h. The total RNA was collected and analyzed by RT-PCR. (**C**) Cells were pretreated without or with DPI (1 μM) for 1 h and then incubated with 10 nM BK for the indicated time intervals or 5 min. The Nox activity was analyzed. (**D**) The total RNA was collected and analyzed Nox isotypes expression by RT-PCR. (**E**) Cells were transfected with scramble (scra) or Nox2 siRNA for 24 h, followed by incubation with 10 nM BK for 24 h. The conditioned media and cell lysates were collected for zymography of MMP-9 or Western blotting to determine the levels of Nox2 and GAPDH (as an internal control). Data are expressed as the mean ± SEM (n = 3). ^*^P < 0.05; ^#^*P* < 0.01, as compared with the respective values of cells stimulated with vehicle (**C**) and BK (**A-C, E**) only. The figure represents one of three similar experiments.

The NADPH oxidase (Nox) is considered to be a major source of ROS in several physiological and pathological processes [8,24]. To explore whether Nox is involved in BK-induced MMP-9 expression, as shown in Figure 2A, B, BK-induced MMP-9 protein and mRNA expression was attenuated by pretreatment with a Nox inhibitor diphenyleneiodonium (DPI, 1 μM). To further investigate whether BK stimulates Nox activity, as shown in Figure 2C, BK time-dependently stimulated Nox activity which was attenuated by pretreatment with DPI (1 μM) in RBA-1 cells. To determine which Nox isoforms involved in these responses, the expression of Nox isoforms was analyzed by RT-PCR. The data showed that Nox1, Nox2, and Nox4 were expressed in RBA-1 cells and Nox2 predominantly expressed in comparison with those of Nox1 and Nox4 mRNA levels (Figure 2D). Next, the involvement of Nox2 in BK-induced responses was confirmed by transfection with Nox2 siRNA. As shown in Figure 2E, transfection of cells with Nox2 siRNA knocked down Nox2 protein expression and attenuated BK-induced MMP-9 expression in RBA-1 cells. These results suggested that BK-induced MMP-9 expression is mediated through Nox(2)-dependent ROS generation in RBA-1 cells.

### Involvement of p47^phox^/Nox2-dependent ROS generation in BK-induced MMP-9 expression

**Figure 3:**
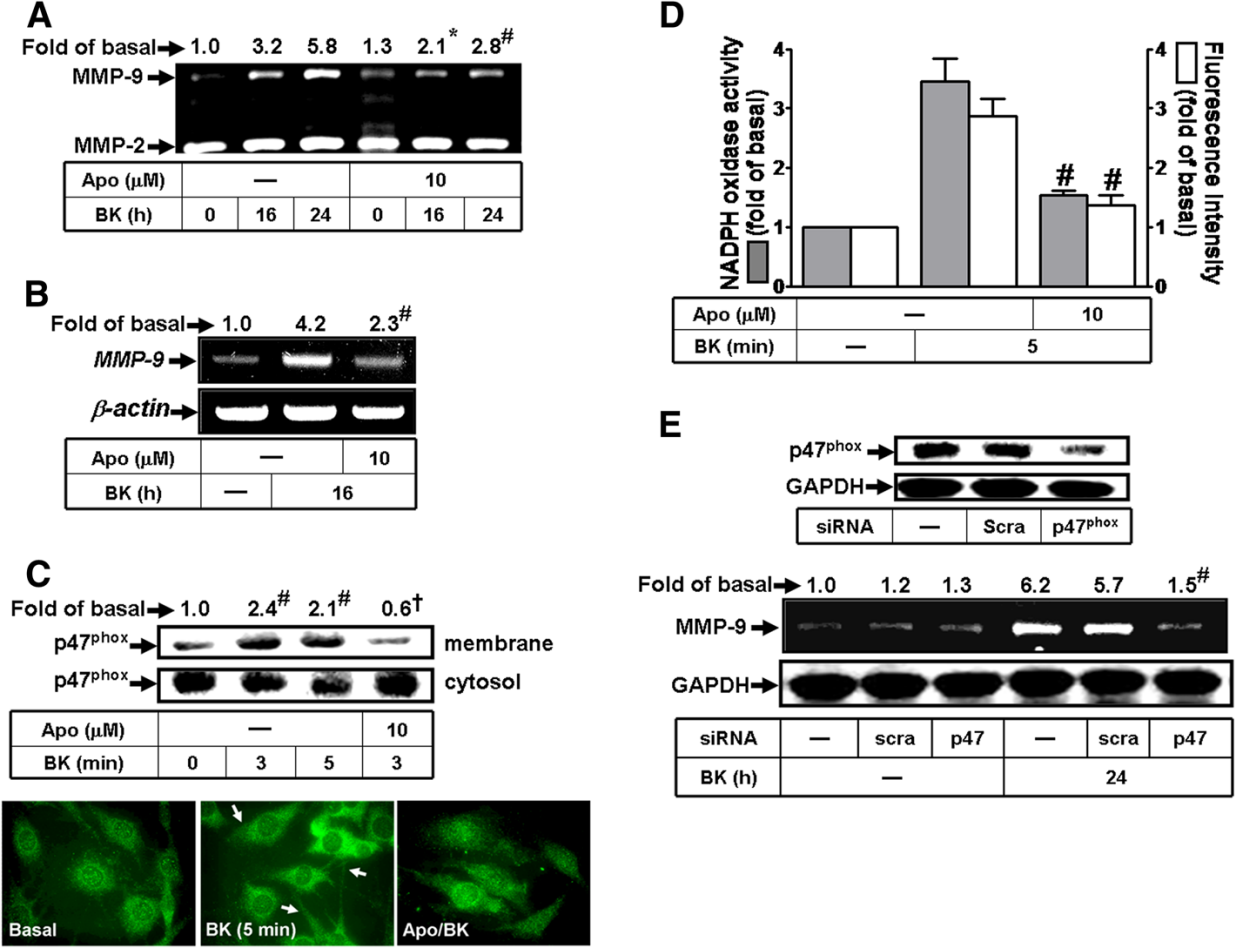
**BK induces MMP-9 expression via p47^phox^ translocation in RBA-1 cells.** (**A**) Cells were pretreated without or with Apocynin (Apo, 10 μM) for 1 h before exposure to 10 nM BK for the indicated time intervals. The conditioned media were collected for zymographic analysis of MMP-9 expression. (**B**) Cells were pretreated with or without Apo (10 μM) for 1 h before exposure to 10 nM BK for 16 h. The total RNA was collected and analyzed by RT-PCR. (**C**) Cells were pretreated without or with Apo (10 μM) for 1 h treated with 10 nM BK for the indicated time intervals or 3 min. The membrane and cytosol fractions were prepared and analyzed by Western blotting. The p47^phox^ translocation was also confirmed by immunofluorescent staining. (**D**) Cells were pretreated without or with Apo (10 μM) for 1 h before exposure to 10 nM BK for 5 min. The Nox activity and ROS generation were analyzed. (**E**) Cells were transfected with scramble (scra) or p47^phox^ siRNA for 24 h, followed by incubation with 10 nM BK for 24 h. The conditioned media and cell lysates were collected for zymography of MMP-9 or Western blotting to determine the levels of Nox2 and GAPDH (as an internal control). Data are expressed as the mean ± SEM (n = 3). ^*^P < 0.05; ^#^*P* < 0.01, as compared with the respective values of cells stimulated with vehicle (**C**) and BK (**A, B, D, E**) only. The figure represents one of three similar experiments.

Nox is a multimeric protein complex consisting of at least three cytosolic subunits of p47^phox^, p67^phox^, and p40^phox^. p47^phox^ has been shown to organize the translocation of other cytosolic factors, hence its designation as “organizer” [25]. Here, to investigate the role of p47^phox^ in BK-induced MMP-9 expression, a p47^phox^ subunit inhibitor apocynin (Apo) was used. The results showed that pretreatment with Apo (10 μM) attenuated BK-induced MMP-9 protein and mRNA expression (Figure 3A, B). We next determined whether the translocation of p47^phox^ involved in BK-induced responses, as shown in Figure 3C, BK stimulated translocation of p47^phox^ from the cytosol to the membrane with a maximal response within 3 min, which was attenuated by pretreatment with Apo. These results were further supported by the data of immunofluorescence images using a fluorescent microscope (Figure 3C). To ascertain that p47^phox^ is essential for BK-stimulated Nox-dependent ROS generation, the Nox activity and ROS generation were detected. As shown in Figure 3D, pretreatment with Apo (10 μM) inhibited the BK-stimulated Nox activation and ROS generation (Figure 3D). To further ensure the effect of p47^phox^ on BK-induced MMP-9 expression, as shown in Figure 3E, transfection with p47^phox^ siRNA significantly knocked down p47^phox^ protein expression and blocked BK-induced MMP-9 expression. These results suggested that p47^phox^/Nox-dependent ROS generation plays a crucial role in BK-induced MMP-9 expression in RBA-1 cells.

### Role of Ca^2+^ in BK-induced ROS generation and MMP-9 expression

**Figure 4:**
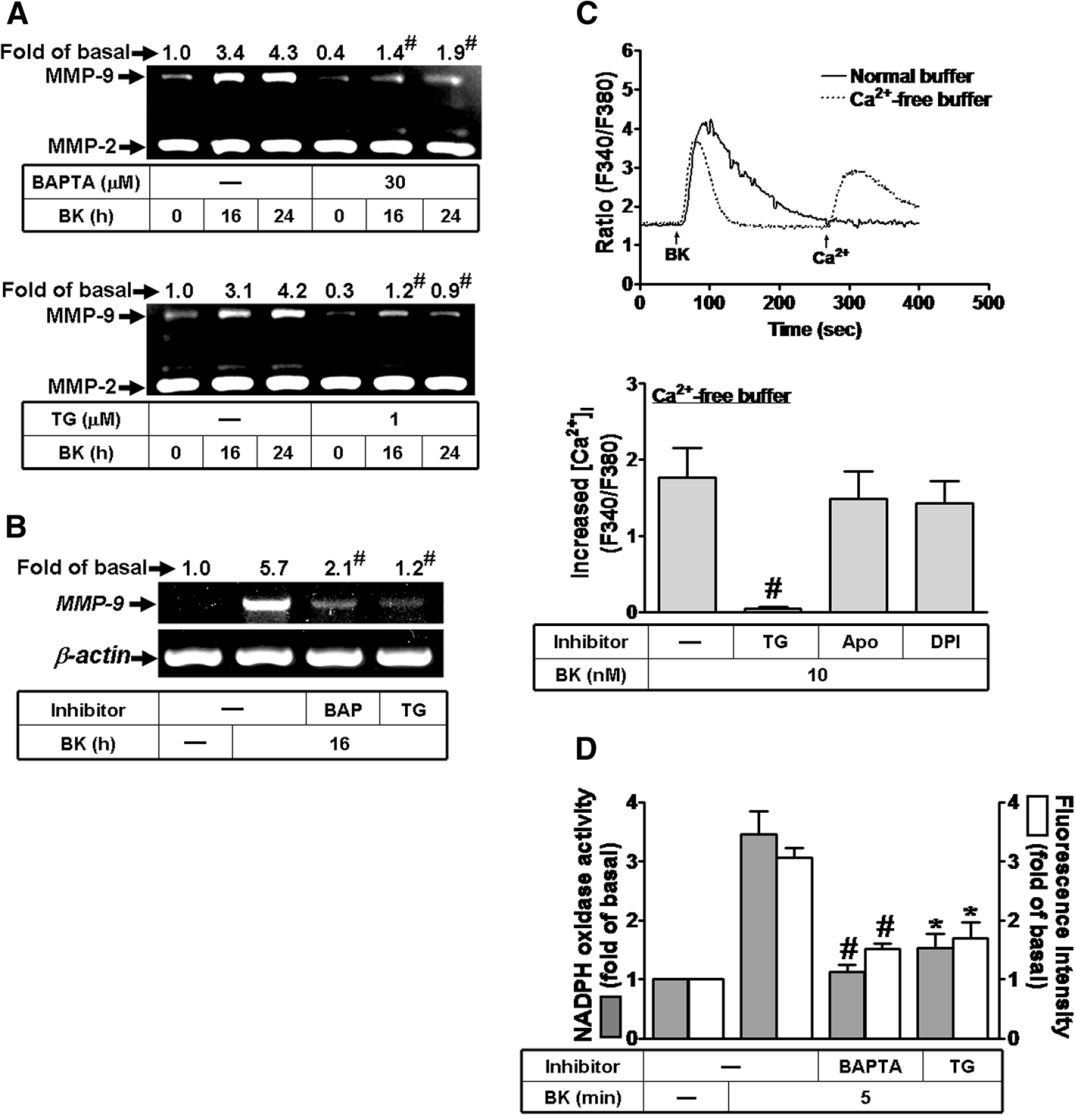
**BK-induced Ca^2+^ release from internal TG-sensitive Ca^2+^ store plays a role in BK-induced MMP-9 expression.** (**A**) Cells were pretreated without or with BAPTA/AM (30 μM) or TG (1 μM) for 1 h before exposure to 10 nM BK for the indicated time intervals. The conditioned media were collected for zymographic analysis of MMP-9 expression. (**B**) Cells were pretreated with or without BAPTA/AM (30 μM) or TG (1 μM) for 1 h before exposure to 10 nM BK for 16 h. The total RNA was collected and analyzed by RT-PCR. (**C**) For Ca^2+^ mobilization, confluent cultures of RBA-1 cells on glass coverslips were loaded with Fura-2/AM and fluorescent measurement of [Ca^2+^]_i_ was carried out in a dual excitation wavelength spectrofluorometer, with excitation at 340 nm and 380 nm. Cells were incubated in Ca^2+^-containing normal buffer (solid line) or Ca^2+^-free buffer (dot line) and then exposed to BK at 50 sec. In a Ca^2+^-free buffer, cells were pretreated with or without TG (1 μM), Apo (10 μM), or DPI (1 μM) for 30 min, exposed to BK at 50 sec, and then 2 mM Ca^2+^ was added to the cells. (**D**) RBA-1 cells were pretreated without or with BAPTA/AM (30 μM) or TG (1 μM) for 1 h and then incubated with 10 nM BK for 5 min. The Nox activity and ROS generation were analyzed. Data are expressed as the mean ± SEM (n = 3). ^*^P < 0.05; ^#^*P* < 0.01, as compared with the respective values of cells stimulated with BK only. The figure represents one of three similar experiments.

Induction of MMP-9 by several stimuli is mediated through Ca^2+^-dependent pathways [21,26]. Therefore, to investigate whether intracellular Ca^2+^ change involves in BK-induced responses, an intracellular Ca^2+^ chelator (BAPTA/AM) and an ER Ca^2+^-ATPase blocker (thapsigargin, TG) were used. Pretreatment with either BAPTA/AM (30 μM) or TG (1 μM) markedly attenuated BK-induced MMP-9 protein and mRNA expression (Figure 4A, B). Next, to determine the role of intracellular Ca^2+^ increase in BK-induced responses, an intracellular Ca^2+^ indicator Fura-2/AM was used. As shown in Figure 4C, BK stimulated a rapid increase in intracellular Ca^2+^ under normal buffer (solid line). To differentiate Ca^2+^ release from intracellular stores or Ca^2+^ influx from the extracellular fluid, the same experiments were performed under Ca^2+^-free buffer. The data showed that BK also stimulated an intracellular Ca^2+^ increase under Ca^2+^-free condition, but smaller than those of normal buffer, which was attenuated by pretreatment with 1 μM TG (Figure 4C, lower panel), suggesting that BK stimulates intracellular Ca^2+^ increase via TG-sensitive intracellular Ca^2+^ stores and extracellular Ca^2+^ influx. We also determined the interplay between Ca^2+^ and Nox/ROS generation in BK-induced MMP-9 expression. The data showed that pretreatment with either Apo (10 μM) or DPI (1 μM) had no effect on BK-stimulated intracellular Ca^2+^ increase (Figure 4C, lower panel). In contrast, pretreatment with either BAPTA/AM (30 μM) or TG (1 μM) significantly attenuated BK-stimulated Nox activity and ROS generation (Figure 4D). These results indicated that intracellular Ca^2+^ increase is essential for BK-induced Nox/ROS-dependent MMP-9 expression in RBA-1 cells.

### BK induces MMP-9 expression via a Ca^2+^-dependent PKC-α manner

**Figure 5:**
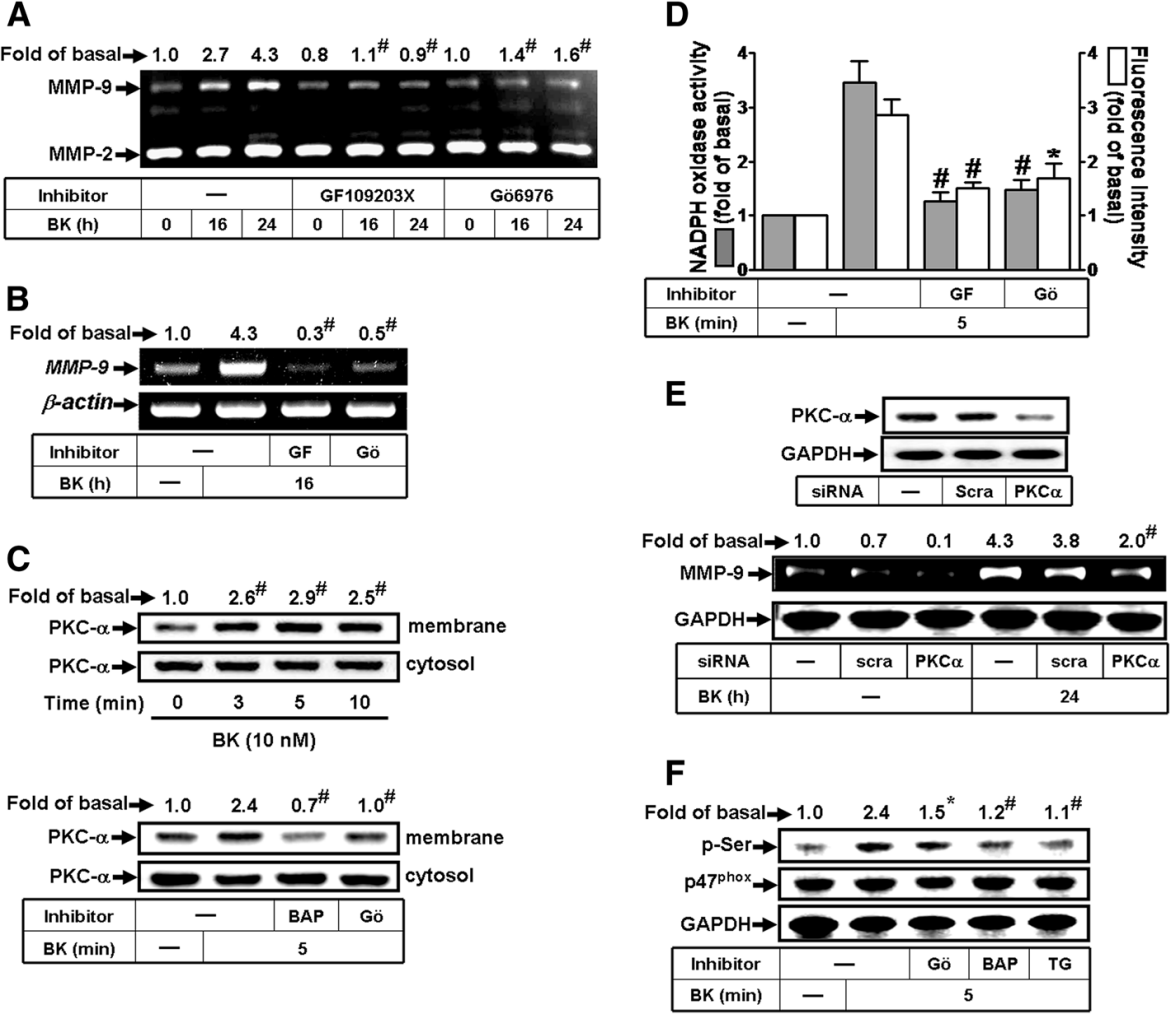
**Involvement of PKC-α in BK-induced ROS generation and MMP-9 expression in RBA-1.** (**A**) Cells were pretreated without or with GF109203X (GF, 30 μM) or Gö6976 (Gö, 1 μM) for 1 h before exposure to 10 nM BK for the indicated time intervals. The conditioned media were collected for zymographic analysis of MMP-9 expression. (**B**) Cells were pretreated with or without GF109203X (GF, 30 μM) or Gö6976 (Gö, 1 μM) for 1 h before exposure to 10 nM BK for 16 h. The total RNA was collected and analyzed by RT-PCR. (**C**) Cells were pretreated with or without BAPTA (BAP, 30 μM) or Gö (1 μM) for 1 h and then treated with 10 nM BK for the indicated time intervals (upper part) or 5 min (lower part). The membrane and cytosol fractions were prepared and analyzed by Western blotting. (**D**) Cells were pretreated without or with GF or Gö for 1 h before exposure to 10 nM BK for 5 min. The Nox activity and ROS generation were analyzed. (**E**) Cells were transfected with scramble (scra) or PKC-α siRNA for 24 h, followed by incubation with 10 nM BK for 24 h. The conditioned media and cell lysates were collected and analyzed by zymography or Western blotting. (**F**) Cells were pretreated without or with Gö (1 μM), BAPTA (30 μM), or TG (1 μM) for 1 h before exposure to 10 nM BK for 5 min. The cell lysates were collected and analyzed by Western blotting using an anti-phospho-serine (p-Ser), p47^phox^, or GAPDH antibody. Data are expressed as the mean ± SEM (n = 3). ^*^P < 0.05; ^#^*P* < 0.01, as compared with the respective values of cells stimulated with vehicle (**C**) and BK (**A, B, D-F**) only. The figure represents one of three similar experiments.

PKC isoforms such as PKC-α have been shown to regulate MMP-9 expression in various cell types [27]. Our recent report also suggested that BK-stimulated Nox/ROS signaling might be mediated through PKC [16]. Thus, to investigate which PKC isoforms are involved in BK-induced resposnes, pretreatment with either a pan-PKC inhibitor GF109203X (30 μM) or a selective PKC-α inhibitor Gö6976 (1 μM) attenuated BK-induced MMP-9 expression (Figure 5A, B), suggesting that PKC(α) is involved in BK-induced MMP-9 expression in RBA-1 cells. Moreover, the activation of PKC-α in BK-induced responses was further confirmed by determining its translocation from the cytosol to the membrane. As shown in Figure 5C, BK stimulated PKC-α translocation in a time-dependent manner with a maximal response within 5 min (Figure 5C, upper part), which was attenuated by pretreatment with either BAPTA/AM (30 μM) or Gö6976 (1 μM) (Figure 5C, lower part). We further examined the role of PKC-α in BK-stimulated Nox-derived ROS generation, pretreatment with GF109203X or Gö6976 attenuated the BK-stimulated Nox activation and ROS generation (Figure 5D), suggesting that PKC(α) is involved in these responses. To further ensure the role of PKC-α in MMP-9 expression, as shown in Figure 5E, ttransfection with PKC-α siRNA knocked down PKC-α protein expression and attenuated BK-induced MMP-9 expression. To determine whether PKC-α is an upstream molecule of p47^phox^ activation, as shown in Figure 5F, pretreatment with Gö6976 (1 μM) significantly attenuated BK-stimulated phosphorylation of p47^phox^ at serine residues. Moreover, pretreatment with either BAPTA/AM (30 μM) or TG (1 μM) also attenuated phosphorylation of p47^phox^ (Figure 5F). These results demonstrated that PKC-α contributes to BK-induced Nox (p47^phox^)/ROS generation and MMP-9 expression in RBA-1 cells.

### Activation of AP-1 is required for BK-induced MMP-9 expression

**Figure 6:**
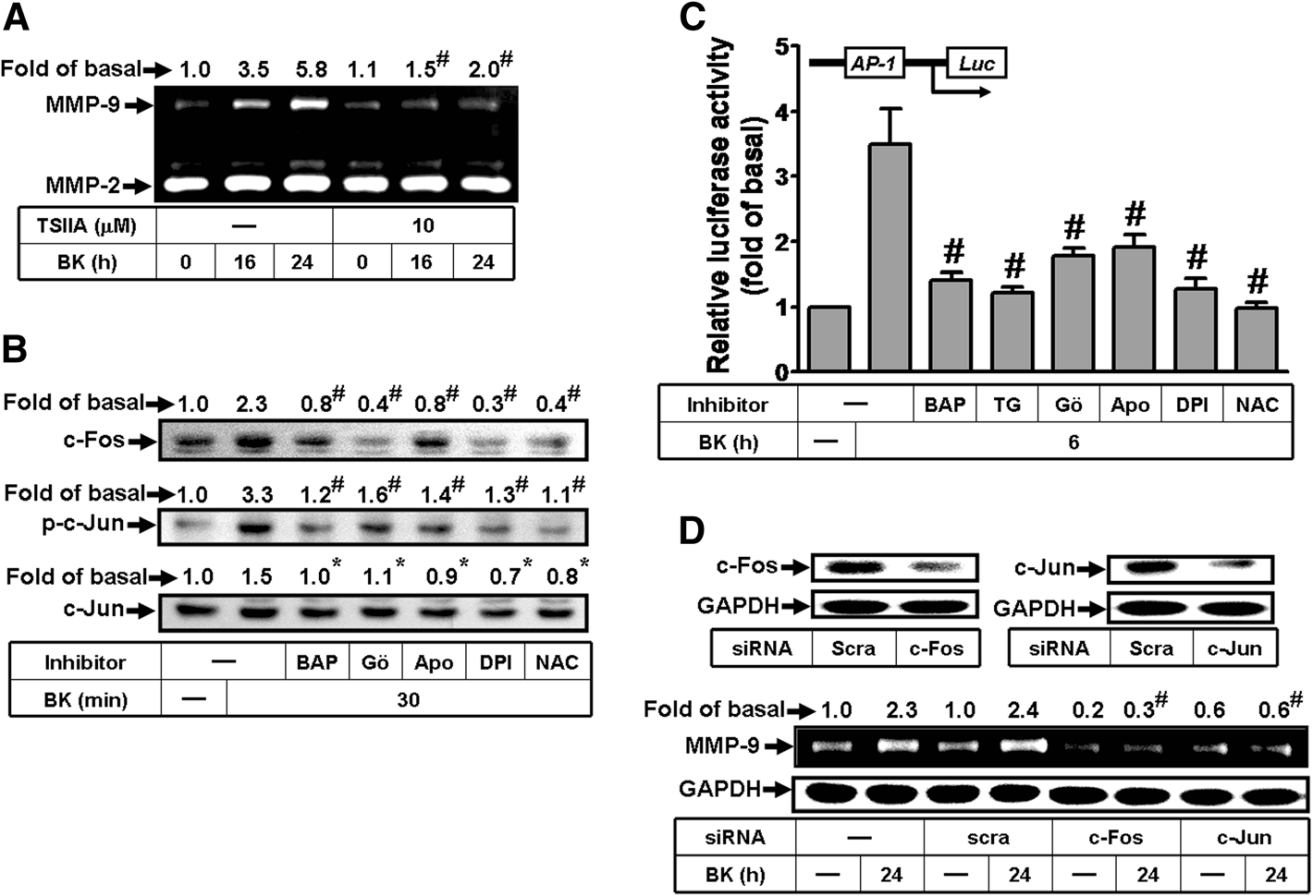
**AP-1 (c-Fos/c-Jun) is essential for BK-induced MMP-9 expression through a Ca^2+^/PKC-α/Nox2/ROS cascade.** (**A**) Cells were pretreated without or with tanshinone IIA (TSIIA, 10 μM) for 1 h before exposure to 10 nM BK for the indicated time intervals. The conditioned media were collected for zymographic analysis of MMP-9 expression. (**B**) Cells were pretreated without or with BAPTA (BAP, 30 μM), Gö6976 (Gö, 1 μM), Apo (10 μM), DPI (1 μM), or NAC (10 mM) for 1 h before exposure to 10 nM BK for 30 min. The nuclear fraction was collected and analyzed by Western blotting using an anti-c-Fos, phospho-c-Jun, or c-Jun antibody. (**C**) Cells were transiently cotransfected with pAP1-Luc and pGal for 24 h, pretreated with BAPTA (BAP), TG, Gö, Apo, DPI, or NAC for 1 h and then incubated with BK for 6 h. The AP-1 promoter activity in the cell lysates was determined. (**D**) Cells were transfected with scramble (scra) or c-Fos/c-Jun siRNA for 24 h, followed by incubation with 10 nM BK for 24 h. The conditioned media and cell lysates were collected and analyzed by zymography or Western blotting. Data are expressed as the mean ± SEM (n = 3). ^*^P < 0.05; ^#^*P* < 0.01, as compared with the respective values of cells stimulated with BK only. The figure represents one of three similar experiments.

AP-1-dependent pathway has been shown to involve in MMP-9 expression in various cell types [22]. To determine whether AP-1 is required for MMP-9 induction by BK, an AP-1 inhibitor (tanshinone IIA) was used. As shown in Figure 6A, BK-induced MMP-9 expression was attenuated by pretreatment with tanshinone IIA (TSIIA, 10 μM). The expression and nuclear accumulation of AP-1 serve as an indicator of downstream gene activation [28]. Thus, we examined whether BK stimulates nuclear accumulation of AP-1 (*i.e.* c-Fos and c-Jun) in RBA-1 cells. First, we found that BK stimulated an accumulation of c-Fos and c-Jun (phospho-c-Jun) in nucleus within 30 min, which was blocked by BAPTA/AM (30 μM), Gö6976 (1 μM), apocynin (10 μM), DPI (1 μM), or NAC (10 mM) (Figure 6B). To determine whether BK enhanced AP-1 transcriptional activity, a promoter containing AP-1 binding sites reporter construct was used. The data showed that BK stimulated an increase in AP-1 promoter activity which was significantly inhibited by pretreatment with BAPTA/AM (30 μM), TG (1 μM), Gö6976 (1 μM), Apo (10 μM), DPI (1 μM), or NAC (10 mM) (Figure 6C). To further confirm the role of AP-1 in BK-induced MMP-9 expression, cells were transfected with c-Fos or c-Jun siRNA. The results showed that transfection with c-Fos or c-Jun siRNA significantly knocked down respective c-Fos or c-Jun proteins and attenuated BK-induced MMP-9 expression (Figure 6D). These results suggested that BK stimulates AP-1 (*i.e.* c-Fos and c-Jun) activation via the PKC-α-dependent Nox2/ROS generation, which is essential for up-regulation of MMP-9 in RBA-1 cells.

### BK enhances recruitment of AP-1 to MMP-9 promoter and astrocytic migration

**Figure 7:**
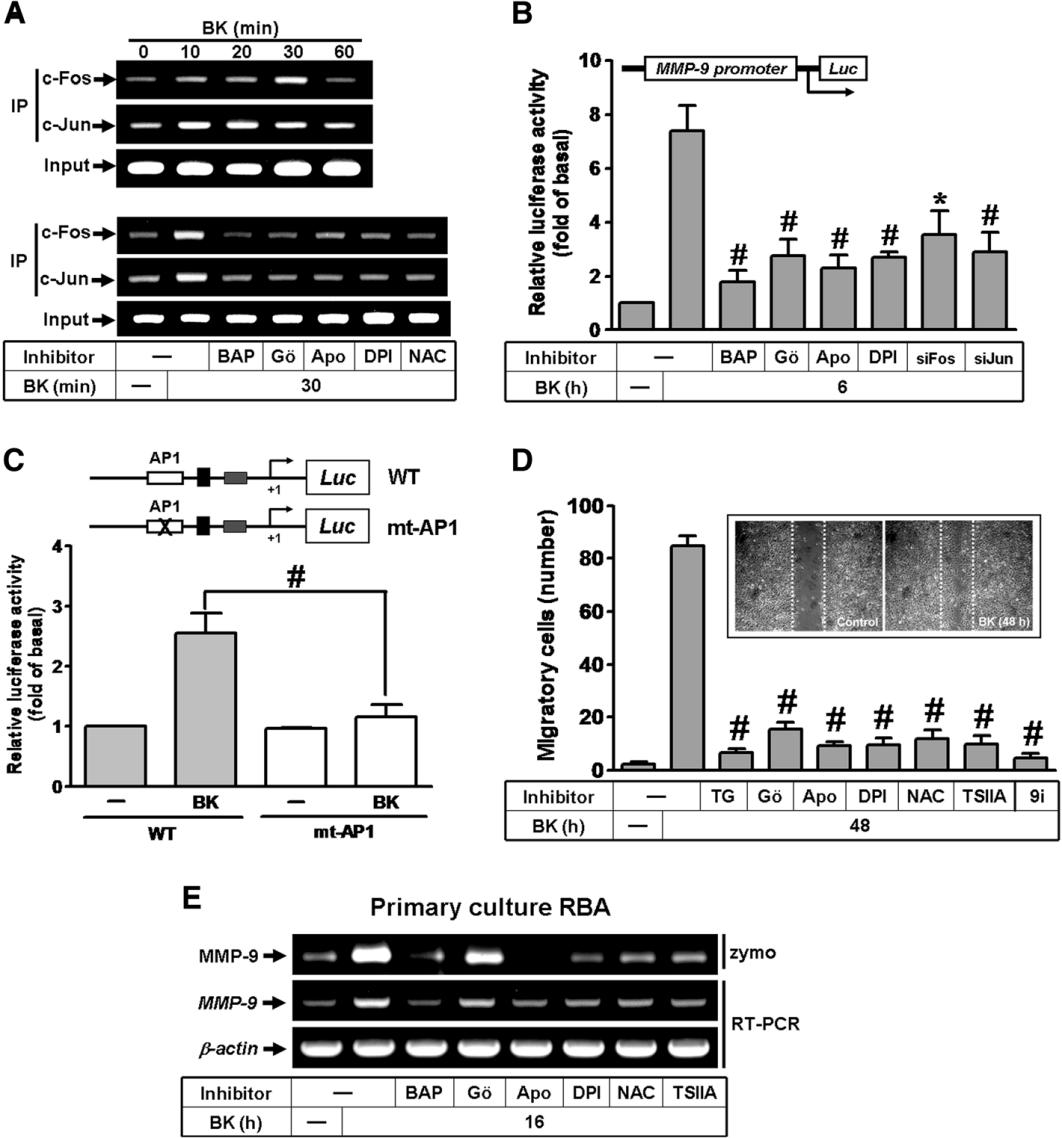
**BK induces astrocytic migration through Nox/ROS-dependent AP-1 increasing MMP-9 gene expression.** (**A**) Cells were incubated with BK (10 nM) for the indicated times (upper part), or pretreated with BAPTA (BAP, 30 μM), Gö6976 (Gö, 1 μM), Apo (10 μM), DPI (1 μM), or NAC (10 mM) for 1 h and then incubated with 10 nM BK for 30 min. The c-Fos and c-Jun AP-1 binding activity were analyzed by chromatin-IP (ChIP)-PCR assay. (**B**) Cells were transiently cotransfected with pGL-MMP9-Luc and pGal for 24 h, pretreated with BAPTA (BAP), Gö, Apo, DPI, or transfection with c-Fos or c-Jun siRNA for 24 h and then incubated with BK for 16 h. (**C**) Schematic representation of the different MMP-9-luciferase constructs, either wild-type (WT) or modified by single-point mutation of the AP-1 binding site (upper part). After cotransfection, luciferase activities of different MMP-9-promoter constructs after stimulation with or without BK (10 nM) for 16 h, were measured as relative MMP-9 promoter activity to that of β-galactosidase. (**D**) Cells were plated on 6-well culture plates, grew to confluence and starved with serum-free DMEM/F-12 medium for 24 h. Cells were pretreated with TG, Gö, Apo, DPI, NAC, TSIIA, or MMP-9i (9i) for 1 h and the monolayer cells were manually scratched with a blue tip as described in Methods, and then incubated with BK (10 nM) for 48 h. Phase contrast images of cells were taken at 48 h and the number of cell migration was counted as described in Methods. (**E**) Rat primary cultured astrocytes were pretreated with BAPTA (BAP), Gö, Apo, DPI, NAC, or TSIIA before exposure to BK for 16 h. The conditioned media and total RNA were collected and analyzed by zymography and RT-PCR. Data are expressed as the mean ± SEM (n = 3). ^#^*P* < 0.01, as compared with the respective values of cells stimulated with BK only.

MMP-9 promoter region contains AP-1 binding sites [29]. Hence, we used ChIP-PCR assay to determine whether BK stimulates recruitment of AP-1 to MMP-9 promoter leading to MMP-9 expression. We designed a pair of primers for MMP-9 promoter (-597 to -318) region, containing an AP-1 binding site. Chromatin was immunoprecipitated using an anti-c-Fos or anti-c-Jun antibody, and the MMP-9 promoter region (-597 to -318) was amplified by PCR. As shown in Figure 7A (upper part), BK time-dependently stimulated binding of c-Fos and c-Jun to the MMP-9 promoter with a maximal response within 30 min, which was attenuated by pretreatment with BAPTA/AM, Gö6976, Apo, DPI, or NAC (Figure 7A, lower part). We next examined whether BK-induced MMP-9 promoter activity is also regulated by these signaling components. We have constructed a MMP-9 promoter into a pGL3-basic vector containing the luciferase reporter system (pGL-MMP-9-Luc), which contains the AP-1 binding sites [30]. BK stimulated an increase in MMP-9 promoter activity which was attenuated by pretreatment with BAPTA/AM, Gö6976, Apo, or DPI and transfection with c-Fos or c-Jun siRNA (Figure 7B), suggesting that BK-induced MMP-9 promoter activity is mediated through PKC-α, Nox/ROS, and AP-1 in RBA-1 cells. To further determine the functional role of AP-1 transcription factor in BK-induced MMP-9 promoter induction, wild-type (WT) and point-mutated (mt)-AP1 MMP-9 promoter constructs (as illustrated in Figure 7C, upper part) were tested for induction by BK. As shown in Figure 7C, BK driven MMP-9 promoter activity was totally lost in AP1 promoter mutant, thus indicating that AP-1 binding domain is required for MMP-9 promoter activation by BK in RBA-1 cells. These results confirmed that BK-increased MMP-9 promoter activity is mediated through binding of AP-1 (c-Fos/c-Jun) to the MMP-9 promoter region.

Ultimately, to demonstrate the functional effect of up-regulated MMP-9 by BK, we evaluated the cell migration of RBA-1 cells. The images of cell migration induced by BK (10 nM) were observed and taken at 48 h (Figure 7D, inset panel). The number of migratory cells was counted and the statistical data were presented in Figure 7D. The data showed that pretreatment with TG (1 μM), Gö6976 (1 μM), Apo (10 μM), DPI (1 μM), NAC (10 mM), TSIIA (10 μM), or MMP-9i (9i, 1 μM) significantly blocked BK-induced cell migration, suggesting that BK-induced MMP-9 expression and cell migration is mediated through Ca^2+^/PKC-α-dependent Nox2/ROS and AP-1 cascade in RBA-1 cells.

To confirm these results in primary culture astrocytes, the rat primary cultured astrocytes were pretreated with BAPTA (BAP), Gö, Apo, DPI, NAC, or TSIIA before exposure to BK (10 nM) for 16 h. The conditioned media and total RNA were collected and analyzed by zymography and RT-PCR analysis. As shown in Figure 7E, pretreatment with BAPTA (BAP), Gö, Apo, DPI, NAC, or TSIIA significantly inhibited BK-induced MMP-9 protein and mRNA expression, suggesting that BK induced MMP-9 expression via Ca^2+^-dependent PKC-α, Nox-derived ROS, and AP-1 pathways in the primary cultured astrocytes.

## Discussion

MMP-9 expression and activation play a critical role in tissue remodeling associated with the pathogenesis of brain diseases [3]. Reduction of MMP activity by pharmacological inhibitors or gene knock-out strategies protects the brain from BBB disruption, cell death, and advanced neuroinflammation [2,29]. Moreover, BK and related peptides are simultaneously produced and released following brain injury [14]. The role of BK implicated in astrocytic functions is not completely understood. Thus, we investigated the molecular mechanisms underlying BK-induced MMP-9 expression in cultured RBA-1 cells and an animal model. Our results suggest that in brain astrocytes, activation of Ca^2+^/PKC-α-dependent Nox2/ROS signal leading to induction of AP-1 (c-Fos/c-Jun) is essential for BK-induced MMP-9 gene expression and enhancing cell migration. The findings suggest that BK-induced MMP-9 expression and cell migration may contribute to increase BBB permeability and recruit immune cells, leading to brain inflammation and edema. In addition, astrocytic migration may be involved in brain inflammation and remodeling during brain injuries such as brain wound healing, tissue remodeling, and glial scar formation [2,3,19,20].

Imbalance in the level of ROS has been shown to play a causative role in numerous pathologies of degenerative diseases [23,31]. ROS concentration-dependently exert a key role in the normal physiological functions and the inflammatory responses [9]. In the brain, ROS also extend to the control of vascular tone which is tightly modulated by metabolic activity within neurons [6]. Moreover, increasing ROS generation by diverse stimuli can regulate the expression of inflammatory mediators in pathogenesis of brain disorders [24,31]. Recently, the cellular damage in neurodegenerative disorders such as Alzheimer’s disease is attributed to oxidative stress [7,10,11]. BK-induced ROS generation has been reported in cerebral arterioles and renal diseases [13]. In this study, our data demonstrated that in both *in vitro* and *in vivo* studies, BK induces MMP-9 expression via ROS-dependent pathways in brain astrocytes. Moreover, we found that BK-induced MMP-9 expression is mediated through Nox-dependent ROS generation, since pretreatment with ROS scavenger NAC or Nox activity inhibitor DPI attenuated BK-induced responses. The involvement of Nox-dependent ROS generation in BK-induced responses was further confirmed by NAC or DPI in RBA-1 cells. ROS exert as a major signaling factor which mediates microglial activation induced by inflammatory mediators, including LPS [12]. Herein we are the first group to establish that Nox-dependent intracellular redox signal (ROS generation) contributes to MMP-9 expression induced by BK in brain astrocytes.

Moreover, Nox is considered to be a major source of ROS in several physiological and pathological processes [8,24]. To date, there are five isoforms of Nox have been discovered, including Nox1-5 [24]. Nox1, Nox2, and Nox4 have been shown to be expressed and are crucial for ROS generation in brain cells [8,32]. First, our data demonstrated that Nox activity is involved in BK-induced responses by a Nox inhibitor DPI (Figure 2A-C). As previous reports, we also found that RBA-1 cells express Nox1, Nox2, and Nox4 (Figure 2D). Next, BK-induced MMP-9 expression is predominantly mediated through activation of Nox2, confirmed by respective Nox siRNAs (Figure 2E). The involvement of Nox in BK-induced responses is mediated through phosphorylation and translocation of p47^phox^ (a Nox2 component) which was attenuated by a p47^phox^ inhibitor apocynin or p47^phox^ siRNA (Figure 3). These results are consistent with previous studies showing that Nox is expressed in astrocytes and contributes to ROS generation [16,33] and Nox is involved in LPS-induced MMPs expression in Raw264.7 [34]. Additionally, we also demonstrated that BK-stimulated Nox2-dependent ROS signal and MMP-9 expression is mediated through Ca^2+^-dependent PKC-α activation, confirmed by an intracellular Ca^2+^ chelator (BAPTA/AM), the inhibitor of ER Ca^2+^-ATPase (TG), pan-PKC (GF109203X), or PKC-α (Gö6976) and PKC-α siRNA (Figure 4, 5). These data demonstrated that BK stimulates Ca^2+^-dependent PKC-α activation linking to Nox2/ROS generation and MMP-9 expression in RBA-1 cells. It is consistent with previous studies indicating that overexpression of PKC-α can increase phosphorylation of p47^phox^ and induce both its translocation and Nox activation in human neutrophils [35].

The excessive increase of oxidative stress during injuries not only causes oxidative damage to cellular macromolecules, but also modulates the pattern of gene expression through functional alterations of transcription factors. The transcription factor AP-1 is well known to be modulated during oxidative stress associated with inflammatory diseases [36]. Moreover, BK-induced gene expression has been shown to be mediated through one of transcription factors such as AP-1 [18]. However, the mechanistic connection between the MMP-9 expression and the ROS-dependent pathway induced by BK has not been established in RBA-1 cells. In this study, we demonstrated that AP-1 is essential for BK-induced MMP-9 expression which was inhibited by an AP-1 inhibitor TSIIA (Figure 6A). The involvement of AP-1 in BK-induced responses was further confirmed by determining induction of AP-1 (c-Fos/c-Jun), including phosphorylation of c-Jun, accumulation of c-Fos in nucleus, and AP-1 promoter reporter activity. These responses were attenuated by the Ca^2+^ chelator (BAPTA/AM), the inhibitor of PKC-α (Gö6976), p47^phox^ (apocynin), Nox (DPI), or a ROS scavenger (NAC), suggesting that the Ca^2+^/PKC-α/p47^phox^/Nox/ROS cascade is involved in activation of AP-1 which contributes to BK-induced MMP-9 expression in RBA-1 cells. Moreover, the role of AP-1 in BK-induced MMP-9 expression was also confirmed by either c-Fos or c-Jun siRNA to inhibit BK-induced MMP-9 expression. These results are consistent with the involvement of AP-1 in MMP-9 expression induced by ox-LDL and LTA in RBA-1 cells [21,22].

To confirm AP-1 indeed binds to the promoter region of MMP-9 gene, the binding activity of AP-1 was determined by a ChIP-PCR assay. In this study, BK stimulated AP-1 (c-Fos and c-Jun) recruitment and activation via Ca^2+^/PKC-α-dependent Nox/ROS cascade and enhancing MMP-9 promoter activity. We further confirmed that AP-1 binding site (-503 to -497) within MMP-9 promoter is required for BK-induced MMP-9 transcriptional activity by using an AP-1-mutated MMP-9 promoter construct. These results demonstrated that activation of AP-1 (either c-Fos or c-Jun) is essential for BK-induced MMP-9 gene expression which is mediated through a Ca^2+^/PKC-α-dependent Nox/ROS signal in RBA-1 cells. It is consistent with previous studies showing that AP-1 is involved in MMP-9 expression in various cell types [22,37].

Several reports have shown the multiple effects of BK and MMP-9 on brain glial cells [30,38]. In brain injury, up-regulation of BK, ROS, and MMP-9 may increase BBB permeability, recruit immune cells infiltrating through BBB into the tissues, and subsequently result in brain inflammation and edema [38]. In this study, we suggested that BK-induced Nox2-mediated ROS signal, MMP-9 expression, and astrocytic migration might be involved in brain inflammation and remodeling during brain injuries. Both ROS [39] and MMP-9 [37] have been reported to play a critical role in cell motility in several cell types. Herein we demonstrated that BK induces MMP-9 expression and cell migration via PKC-α-dependent Nox/ROS signaling pathway in RBA-1 cells. The results are consistent with recent reports indicating that the ROS-dependent MMP-9 expression is essential for cell migration by various stimuli such as TGF-β1 or LPS [20,34]. Hence, we suggest that ROS-mediated MMP-9 up-regulation by BK is associated with cell migration in RBA-1 cells. Taken together, BK, ROS, and MMP-9 may have multiple effects on different types of brain glial cells, inducing inflammatory and remodeling roles.

## Conclusions

**Figure 8:**
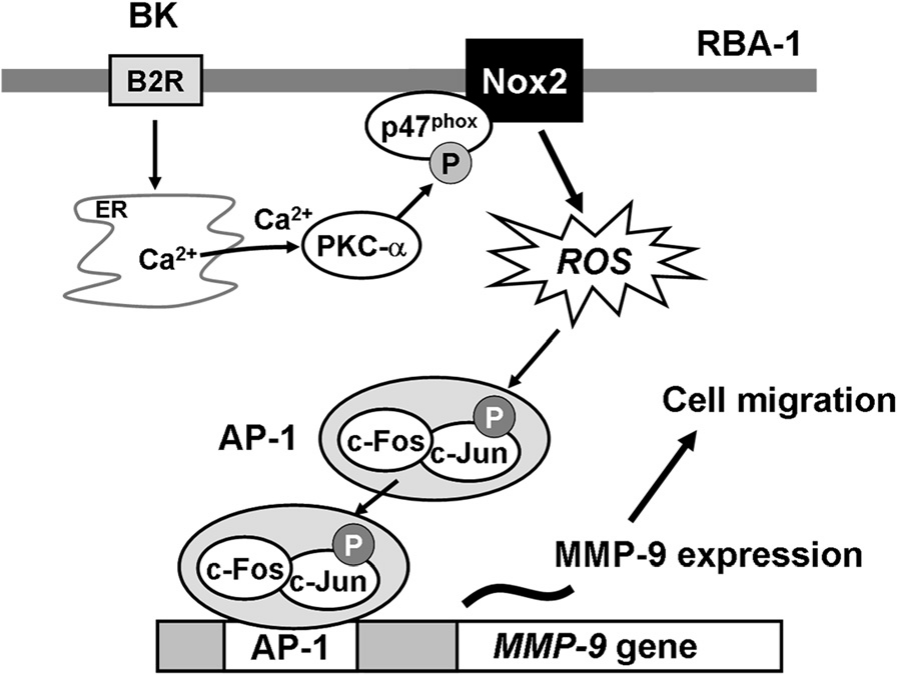
**Scheme of the BK-mediated signals linked to MMP-9 expression and brain astrocytic migration.** Binding of BK to its specific receptors (B2R) stimulates activation of Ca^2+^/PKC-α/Nox2/ROS cascade. MMP-9 transcription is AP-1-dependently regulated by an ROS-dependent pathway. This signaling pathway may contribute to sustained expression of MMP-9 which is required for cell migration in brain astrocytes (RBA-1 cells).

We have demonstrated that BK directly induces MMP-9 expression via Ca^2+^-dependent PKC-α, p47^phox^/Nox2-mediated ROS generation, linking to activation of AP-1 (c-Fos/c-Jun), which results in the cell migration in RBA-1 cells. Based on the observations from literatures and our findings, Figure 8 depicts a model for the molecular mechanisms underlying BK-induced MMP-9 expression and functional changes (*e.g.* migration) in RBA-1 cells. These findings concerning BK-induced Nox-dependent redox signal and MMP-9 up-regulation in brain astrocytes imply that BK-mediated ROS signals might play a critical role in the modulation of brain injuries and inflammatory diseases.

## Methods

### Materials

Dulbecco’s modified Eagle’s medium (DMEM)/Ham’s nutrient mixture F-12 (F-12), fetal bovine serum (FBS), and PKC-α, p47^phox^, Nox2, c-Fos and c-Jun siRNAs were purchased from Invitrogen (Carlsbad, CA). Hybond C membrane and enhanced chemiluminescence (ECL) detection system were from GE Healthcare Biosciences (Buckinghamshire, UK). MMP-9 antibody was from NeoMarker (Fremont, CA). PKC-α, p47^phox^, Nox1, Nox2, Nox4, phospho-c-Jun, c-Fos, and c-Jun antibodies were from Santa Cruz (Santa Cruz, CA). All primary antibodies were diluted at 1:1000 in PBS with 1% BSA. BAPTA/AM, thapsigargin, GF109203X, Gö6976, apocynin, diphenyleneiodonium chloride (DPI), and tanshinone IIA (TSIIA) were from Biomol (Plymouth Meeting, PA). Bicinchoninic acid (BCA) protein assay reagent was from Pierce (Rockford, IL). BK, N-acetyl cysteine (NAC), enzymes, and other chemicals were from Sigma (St. Louis, MO).

### Cell cultures and treatment

The RBA-1 originated from a primary cultured astrocyte of neonatal rat cerebrum and was naturally developed through successive cell passages [30] and used throughout this study. Primary astrocyte cultures were prepared from the cortex of 6-day-old Sprague-Dawley rat pups [30]. The purity of primary cultured astrocytes was assessed using an astrocyte-specific marker, glial fibrillary acidic protein (GFAP), showing over 95% GFAP-positive astrocytes. Cells were plated onto 12-well culture plates and made quiescent at confluence by incubation in serum-free DMEM/F-12 for 24 h, and then incubated with BK at 37°C for the indicated time intervals. When the inhibitors were used, cells were pretreated with the inhibitor for 1 h before exposure to BK.

### Animal model and experimental procedures

All animals were treated in accordance with Chang Gung University guidelines, and the animal protocols were approved by Chang Gung University’s Administrative Panel on Laboratory Animal Care. Male Sprague-Dawley rats (250-300 g) were anesthetized with Pentothal (30 mg/kg, *i.p.*) and placed in a stereotactic frame. A 30-gauge needle attached to a 1.0-ml Hamilton microsyringe was inserted into the cortex via a polyethylene tube (4.0 mm lateral to the midline, 2.0 mm anterior to the coronal suture of the bregma, 2.5 mm below the surface of the skull, and then withdrawn 0.5 mm), and 30 μl of BK (1 μM) was injected over 1 minute using a microsyringe pump. As a control experiment, 30 μl of sterile 0.1% BSA was infused in the same way. After infusion, the needle was slowly removed. The burr hole was sealed with bone wax and the incision was closed. Rectal temperature was controlled within the normal range (37±5°C) during surgery with a homeothermic blanket. The animals were maintained at 25°C with ad libitum access to food and water. To evaluate the involvement of ROS in BK-induced MMP-9 expression, the rats were pretreated with a ROS scavenger NAC (150 mg/kg, i.v.) for 1 h before the BK injection for 24 h. To examine the MMP-9 expression and astrocytes’ localization in brain cortex, immunofluorescence staining was performed on the first and second serial sections of the brain, which were deparaffinized, rehydrated, and washed with PBS. Non-specific binding was blocked by preincubation with PBS containing 5% BSA for 1 h at room temperature. The sections were incubated with an anti-MMP-9 or anti-GFAP antibody for the positive localization and identification of astrocytes for overnight at 4°C. The sections were washed and then incubated with a fluorescein isothiocyanate (FITC)-conjugated goat anti-mouse or anti-rabbit secondary antibody and observed by using a fluorescence microscope (ZEISS, Axiovert 200 M), 400×.

### MMP gelatin zymography

Growth-arrested cells were incubated with BK for the indicated time intervals. Treatment of RBA-1 cells with pharmacological inhibitors or BK alone had no significant effect on cell viability determined by an XTT assay (data not shown). The cultured media were analyzed by gelatin zymography [19]. Gelatinolytic activity was manifested as horizontal white bands on a blue background. Because cleaved MMPs were not reliably detectable, only pro-form zymogens were quantified.

### Total RNA extraction and reverse transcription-PCR analysis

Total RNA was extracted from RBA-1 cells [19]. The cDNA obtained from 0.5 μg total RNA was used as a template for PCR amplification. Oligonucleotide primers were designed on the basis of Genbank entries for rat MMP-9, Nox1-4 [17] and β-actin. The following primers were used for amplification reaction: MMP-9, 5’-AGTTTGGTGTCGCGGAGCAC-3’ (sense), 5’-TACATGAGCGCTTCCGGCAC-3’ (antisense); Nox1, 5’-TACGAAGTGGCTGTACTGGTTG-3’ (sense), 5’-CTCCCAAAGGAGGTTTTCTGTT-3’ (antisense); Nox2, 5’-TCAAGTGTCCCCAGGTATCC-3’ (sense), 5’-CTTCACTGGCTGTACCAAAGG-3’ (antisense); Nox3, 5’-AATCACAGAGTCTGCCTGGACT-3’ (sense), 5’-ATCCAGACTTTCATCCCAGTGT-3’ (antisense); Nox4, 5’-GGAAGTCCATTTGAGGAGTCAC-3’ (sense), 5’-TGGATGTTCACAAAGTCAGGTC-3’ (antisense); β-actin, 5’-GAACCCTAAGGCCAACCGTG-3’ (sense), 5’-TGGCATAGAGGTCTTTACGG-3’ (antisense).

The amplification was performed in 30 cycles at 55°C, 30 s; 72°C, 1 min; 94°C, 30 s. PCR fragments were analyzed on 2% agarose in 1X TAE gel containing ethidium bromide and their sizes were compared to molecular weight markers. Amplification of β-actin, a relatively invariant internal reference RNA, was performed in parallel, and cDNA amounts were standardized to equivalent β-actin mRNA levels. These primer sets specifically recognize only the genes of interest as indicated by amplification of a single band of the expected size (754 bp for MMP-9, 337 bp for Nox1, 209 bp for Nox2, 214 bp for Nox3, 244 bp for Nox4, and 514 bp for β-actin).

### Preparation of cell extracts and Western blotting analysis

Growth-arrested cells were incubated with BK for the indicated time intervals. The cell lysates were collected and the protein concentration was determined by the BCA reagents according to the instructions of the manufacturer. Samples from these cell lysates (30 μg protein) were denatured and subjected to SDS-PAGE using a 10% (w/v) running gel. The phosphorylation of p47^phox^ or c-Jun was analyzed by Western blot [19]. The immunoreactive bands were detected by ECL reagents.

### Measurement of intracellular ROS generation

The peroxide-sensitive fluorescent probe 2’,7’-dichlorofluorescein diacetate (DCF-DA) was used to assess the generation of intracellular ROS [40] with minor modifications. RBA-1 cells on monolayers were incubated with 5 μM DCF-DA in RPMI-1640 for 45 min at 37°C. The supernatant was removed and replaced with fresh RPMI-1640 medium before exposure to BK (10 nM). Relative fluorescence intensity was recorded over time (3-120 min) by using a fluorescent plate reader (Thermo, Appliskan) at an excitation wavelength of 485 nm and emission was measured at a wavelength of 530 nm, and fluorescent images were also obtained by using a fluorescence microscopy (ZEISS, Axiovert 200 M).

### Determination of NADPH oxidase activity by chemiluminescence assay

The NADPH oxidase activity in intact cells was assayed by lucigenin chemiluminescence [41]. After incubation, the cells were gently scraped and centrifuged at 400 × g for 10 min at 4°C. The cell pellet was resuspended in a known volume (35 μl/well) of ice-cold RPMI 1640 medium, and the cell suspension was kept on ice. To a final 200 μl of pre-warmed (37°C) RPMI 1640 medium containing either NADPH (1 μM) or lucigenin (20 μM), 5 μl of cell suspension (2 × 10^4^ cells) was added to initiate the reaction followed by immediate measurement of chemiluminescence using an Appliskan luminometer (Thermo®) in an out-of-coincidence mode. Appropriate blanks and controls were established, and chemiluminescence was recorded. Neither NADPH nor NADH enhanced the background chemiluminescence of lucigenin alone (30-40 counts/min). Chemiluminescence was continuously measured for 12 min, and the activity of NADPH oxidase was expressed as counts per million cells.

### Transient transfection with siRNAs

Transient transfection of small interfering RNA (siRNA) duplexes corresponding to rat PKC-α, p47^phox^, Nox2, c-Fos, c-Jun, and scrambled siRNAs (100 nM) was performed using a Lipofetamine^TM^ RNAiMAX reagent (Invitrogen) according to the manufacturer’s instructions.

### Plasmid construction, transfection, and luciferase reporter gene assays

The upstream region (-1280 to +19) of the rat MMP-9 promoter was cloned to the pGL3-basic vector containing the luciferase reporter system [30]. Additionally, the introduction of a mismatched primer mutation into the AP-1 to generate pGL3-MMP-9ΔAP-1 was performed, using the following (forward) primer: ΔAP-1: 5’-GCAGGAGAGGAAGCTGAGTTGAAGACA-3’. All plasmids were prepared by using QIAGEN plasmid DNA preparation kits. These constructs were transfected into RBA-1 cells by using a Lipofectamine reagent according to the instructions of manufacture. The transfection efficiency (~60%) was determined by transfection with enhanced GFP. After incubation with BK, cells were collected and disrupted by sonication in lysis buffer (25 mM Tris, pH 7.8, 2 mM EDTA, 1% Triton X-100, and 10% glycerol). After centrifugation, aliquots of the supernatants were tested for promoter activity using a luciferase assay system (Promega, Madison, WI). Firefly luciferase activities were standardized for β-galactosidase activity.

### Isolation of cell fractions

Cells were harvested, sonicated for 5 s at output 1.5 with a sonicator (Misonix, Inc., Farmingdale, NY), and centrifuged at 8,000 rpm for 15 min at 4°C. The pellet was collected as the nuclear fraction. The supernatant was centrifuged at 14,000 rpm for 60 min at 4°C to yield the pellet (membrane fraction) and the supernatant (cytosolic fraction).

### Measurement of intracellular Ca^2+^ level

Intracellular Ca^2+^ signaling was measured in confluent monolayers with a calcium-sensitive dye Fura-2/AM as described by Grynkiewicz et al. [42]. Upon confluence, the cells were cultured in serum-free DMEM/F-12 for 24 h before measurements were made. The Ca^2+^ level was determined using Fura-2/AM as an indicator in a temperature-controlled holder of a Hitachi F-4500 spectrofluorometer (Tokyo, Japan), as previously described [19]. The autofluorescence of each monolayer was subtracted from the fluorescent data. The ratios (R) of the fluorescence at the two wavelengths are computed and used to calculate changes in intracellular Ca^2+^ level.

### Immunofluorescence staining

Growth-arrested cells were treated with 10 nM BK for the indicated time intervals, washed twice with ice-cold PBS, fixed with 4% (w/v) paraformaldehyde in PBS for 30 min, and then permeabilized with 0.3% Triton X-100 in PBS for 15 min. The staining was performed by incubating with 10% normal goat serum in PBS for 30 min followed by incubating with an anti-p47^phox^ antibody (1:200 dilution) for 1 h in PBS with 1% BSA, washing thrice with PBS, incubating for 1 h with a FITC-conjugated goat anti-rabbit antibody (1:200 dilution) in PBS with 1% BSA, washing thrice with PBS, and finally mounting with aqueous mounting medium. The images observed under a fluorescence microscope (ZEISS, Axiovert 200 M).

### Chromatin immunoprecipitation assay

To detect the *in vivo* association of nuclear proteins with rat MMP-9 promoter, chromatin immunoprecipitation (ChIP) analysis was conducted as previously described [30]. Briefly, RBA-1 cells were cross-linked with 1% formaldehyde for 10 min at 37°C and washed thrice with ice-cold PBS containing 1 mM phenylmethylsulfonyl fluoride (PMSF) and 1% aprotinin. Soluble chromatin was prepared using a ChIP assay kit (Upstate) according to the manufacturer’s recommendations and immunoprecipitated without (control) or with anti-c-Fos or anti-c-Jun antibody and normal goat immunoglobulin G (IgG). Following washes and elution, precipitates were heated overnight at 65°C to reverse cross-linking of DNA and protein. DNA fragments were purified by phenol-chloroform extraction and ethanol precipitation. The purified DNA was subjected to PCR amplification using the primers specific for the region (-597 to -318) containing the distal AP-1 binding site (-503 to -497) present in the MMP-9 promoter region, sense primer: 5’-AGAGCCTGCTCCCAGAGGGC-3’; antisense primer: 5’-GCCAAGTCAGGCAGGACCCC-3’. PCR fragments were analyzed on 2% agarose in 1X TAE gel containing ethidium bromide and the size (279 bp) was compared to a molecular weight marker.

### Cell migration assay

RBA-1 cells were cultured to confluence in 6-well culture plates and starved with serum-free DMEM/F-12 medium for 24 h. The monolayer cells were manually scratched with a pipette blue tip to create extended and definite scratches in the center of the dishes with a bright and clear field (~2 mm). The detached cells were removed by washing the cells once with PBS. Serum-free DMEM/F-12 medium with or without BK (10 nM) was added to each dish as indicated after pretreatment with the inhibitors for 1 h, containing a DNA synthesis inhibitor hydroxyurea (10 μM) during the period of experiment. Images of migratory cells from the scratched boundary were observed under a light microscope with a digital camera (Olympus, Japan). Numbers of migratory cells were counted from the resulting four phase images for each point and then averaged for each experimental condition. The data presented are summarized from three separate assays.

### Statistical analysis of data

All the data were estimated using a GraphPad Prism Program (GraphPad, San Diego, CA). Data were expressed as the mean±SEM and analyzed by one-way analysis of variance followed with Tukey’s posthoc test. *P* < 0.05 was considered significant.

## Abbreviations

CNS: Central nervous system; BK: Bradykinin; MMP-9: Matrix metalloproteinase-9; RBA-1: Rat brain astrocytes; DMEM/F-12: Dulbecco’s modified Eagle’s medium/Ham’s nutrient mixture F-12; FBS: Fetal bovine serum; ECL: Enhanced chemiluminescence; BCA: Bicinchoninic acid; PBS: Phosphate-buffered saline; GFAP: Glial fibrillary acid protein; GPCR: G protein-coupled receptor; NAC: N-acetyl cysteine; DPI: Diphenylene iodonium; TG: Thapsigargin; PKC-α: Protein kinase C-α; Nox2: NADPH oxidase 2; DCF-DA: 2’,7’-dichlorodihydrofluorescin diacetate; ROS: Reactive oxygen species; siRNA: Small interfering RNA; TSIIA: Tanshinone IIA; RT-PCR: Reverse transcription-polymerase chain reaction; ChIP: Chromatin immunoprecipitation; AP-1: Activator protein 1.

## Competing interests

The authors declare that they have no competing interests.

## Authors’ contributions

CCL, RHS, PLC, and SEC designed and performed experiments, acquisition and analysis of data, and drafted the manuscript. RHS and JCC helped to perform experiments and prepare the manuscript. HLH and CMY have conceived of the study, participated in its design and coordination, CMY has been involved in drafting the manuscript and revising it critically for important intellectual content and has given final approval of the version to be published. All authors read and approved the final version of manuscript.
